# Dry Eye in Diabetes: The Indian Diabetic and Endocrine Eye Diseases (INDEED) Review

**DOI:** 10.17925/EE.2024.20.2.6

**Published:** 2024-10-14

**Authors:** Sanjay Kalra, Nikhil Sharad Gokhale, Ganapathi Bantwal, Roopashri Matada, Shehla Shaikh, Varsha Pawar, Maneesha Khalse, Kamlesh Patel

**Affiliations:** 1. Department of Endocrinology, Bharti Hospital, Karnal, Haryana, India; 2. Gokhale Eye Hospital, Mumbai, India; 3. Department of Endocrinology, St. Johns Medical College, Bangalore, Karnataka, India; 4. Department of Ophthalmology, JJM Medical College, Davangere, Karnataka, India; 5. Saifee Hospital, Mumbai, Maharashtra, India; 6. Medical Affairs Division, Lupin Ltd., Mumbai, Maharashtra, India; 7. Medical and Health Tech, Lupin Ltd., Mumbai, Maharashtra, India

**Keywords:** Epithelial protection, glycaemic control, insulin, lid hygiene, metformin, quality of life, screening, steroids, tear deficiency, tear substitutes

## Abstract

Dry eye disease (DED) is an inadequately addressed condition in the diabetes management process and can significantly impact the quality of life and self-care. Therefore, it was imperative to review DED in the diabetic population. The aim of this article was to obtain insights into the correlation between dry eye and diabetes, with a focus on data published in the Indian population. A comprehensive literature review was performed using MEDLINE and Google Scholar, along with an internet-based search of publicly available information and peer-reviewed publications that may not have been indexed in these databases. The recommendations from several important societies for patients with DED have also been reviewed. Major aspects commonly associated with DED and diabetes have been addressed, and specific suggestions for screening, diagnosis and treatment have been described. Therefore, this review could be an invaluable resource for doctors managing patients with both conditions.

Dry eye disease (DED) is known as dry eye syndrome (DES) or keratoconjunctivitis sicca. According to the Tear Film and Ocular Surface Society’s Dry Eye Workshop II (TFOS DEWS II), it constitutes a multifactorial disease of the ocular surface, characterized by a loss of homeostasis of the tear film and accompanied by ocular symptoms.^1,2^ It may cause ocular discomfort and/or visual symptoms and inflammatory disease of the ocular surface. DED, similar to other ocular conditions such as diabetic retinopathy (DR), papillopathy, cataract and glaucoma, is associated with high morbidity. It negatively affects the quality of life, results in difficulties in performing daily activities and also reduces productivity at work.^3–5^

DED is an important clinical condition, found in a large proportion of people with diabetes; it is reported in 18–54% of patients with diabetes across India.^6^ India is home to 101 million patients with diabetes, nearly 67% of whom suffer from poor glycaemic control.^7,8^ DED is an inadequately addressed condition in the diabetes management process, and the awareness among physicians regarding it is low. DED may impact self-care in patients with diabetes – it may interfere with self-confidence, physical activity and self-administration of injectable glucose-l owering drugs.^9^ Therefore, it was found imperative to address DED in patients with diabetes.^5^

This review aims to obtain insights into the correlation between dry eye and diabetes, with a focus on data published in the Indian population. The aim was to address major aspects commonly associated with DED and diabetes and make specific suggestions for the management of DED in this population so that this review could be an invaluable resource for doctors managing patients with both conditions. A comprehensive literature review was performed using MEDLINE and Google Scholar, along with an internet-based search of publicly available information and peer-reviewed publications that may not have been indexed in these databases. The recommendations from several important societies for patients with DED have also been reviewed.

## Prevalence, risk factors and pathogenesis of dry eye disease in diabetes

The worldwide prevalence studies have reported a range of 15.0%–54.3% DED in people with diabetes.^10,11^ Symptomatic or asymptomatic DED is present in at least half of the people with diabetes mellitus (DM) globally, which is nearly fivefold higher than the number of patients with DED and no DM.^12^ The proportion of patients with symptomatic DED is also twice as high in those with diabetes.^13^ Several studies from India have reported a significantly higher prevalence of DED in people with diabetes than those without.^14–16^ The eyes are exposed organs and therefore influenced by climatic and environmental factors.^17^ Previous studies have found that climatic and environmental changes have differential adverse impacts on dry eyes and likely occur in tropical countries where sunlight and wind exposure are immense.^18^

**Table 1:** Risk factors for dry eye disease in diabetes^27^

Intrinsic factors	Extrinsic factors
Female gender (including post-menopausal status)	Environmental factors, such as poor humidity, high temperatures, pollution, and excessive screen time for reading
Advancing age	Psychological factors, such as depression and stress
Duration of diabetes	
Poor glycaemic control	
Diabetic retinopathy and interventions to treat it	
Pre-existing comorbidities, such as chronic viral infections and Parkinson's disease	
Concomitant use of anticholinergic medications, beta-blockers, oestrogen, interferons and chemotherapy	
Ocular surgeries	
Blepharitis	

The prevalence of DED in patients with type 2 diabetes (T2D) has been reported to be between 15 and 33% in people older than 65 years, and 20% in those aged 43–86 years.^19^ Among children and adolescents with type 1 diabetes, the 3-year incidence rate was 22.5%.^20^ In a study from India, the age-adjusted prevalence of DED in patients with T2D was 18.4% and 23.3% in males and females, respectively.^21^

Population-based studies have shown that poor glycaemic control has also been associated with severe DED symptoms.^22,23^ A significant correlation between the severity of DED and the duration of diabetes has also been reported.^24^ The prevalence of mild DED was ~12% in those with 5–10 years, 37–39% in 11–20 years and ~43% in >20 years of T2D diagnosis.^24^ However, it has been argued that the symptoms of DED are less severe in patients with prolonged disease due to reduced corneal sensitivity associated with diabetic peripheral corneal neuropathy.^25^ The presence of retinopathy or macular oedema doubles the odds of DED.^26,27^ Ocular surgeries for cataract and retinopathy are also associated with DED.^28^

A meta-analysis of four studies including the data from more than two million persons confirmed a significant association between DM and the risk of DED.^29^ Risk factors for DED in the general population also apply to people with diabetes and may increase the cumulative risk (*Table 1*).^27,30^

**Figure 1:**
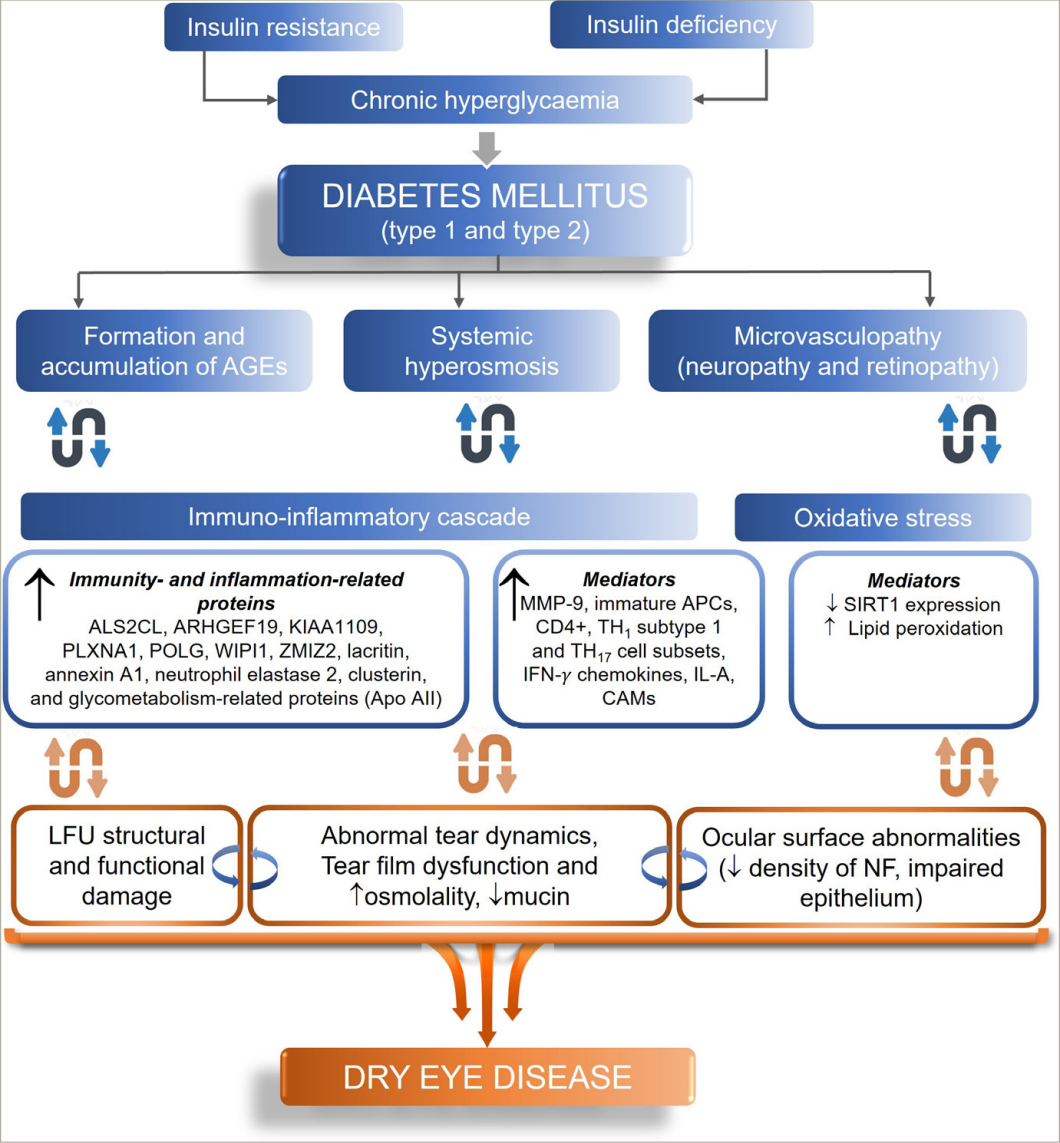
Pathogenesis of dry eye disease in patients with diabetes^32^

Insulin deficiency and consequent hyperglycaemia lead to histological abnormalities in the various ocular components that increase the risk of DED.^30,31^ Insulin resistance or deficiency and chronic hyperglycaemia may reduce the meibomian gland epithelial cells and goblet cells, leading to a deficient tear film.^6^ Moreover, T2D leads to structural anomalies in the corneal nerve fibres, which may also result in decreased sensitivity.^6^ Recent evidence from a cross-sectional study has demonstrated impaired meibomian gland and tear function in patients with T2D, which deteriorated with moderate or long diabetic duration and a high glycated haemoglobin (HbA1c) level. The values of meibomian gland parameters and the corneal fluorescein staining score were significantly lower in patients with T2D without DED than those with T2D with DED. Thus, asymptomatic Meibomian gland dysfunction (MGD) may occur before the ocular discomfort, and DED develops in patients with T2D (*Figure 1*).^31,32^

## Clinical manifestations of dry eye disease in diabetes

Patients with DED often display signs and symptoms of ocular discomfort, such as burning sensation, photopsia, foreign body sensation, soreness, itchiness, redness and blurred vision.^13^ It may cause severe irritation to the ocular surface, predominantly the cornea, causing corneal complications.^13^ Studies have demonstrated a direct association between a higher grade of DED in those with DM compared with those without and more severe signs and symptoms in those with poor glycaemic control (high A1c) versus those with normoglycaemia.^14^ Thus, DM and DED increase the risk of corneal infection, scarring, perforation and irreparable tissue injury.^32^

In both Caucasians and Asians, the degree of DED severity scores was found to be higher, and corneal sensitivity poorer, in patients with diabetes than those without.^33^ A recently published study from India also demonstrated that corneal nerve sensitivity was reduced more in patients with diabetes and moderate DED.^6^ Histological evaluation showed a greater extent of ocular tissue abnormalities in patients with diabetes and DED than in those with DED alone.^33,34^ Prospective, controlled studies comparing age-and gender-matched patients have found approximately twofold higher frequency of DED symptoms in patients with diabetes than those without.^33,35^ A recent meta-analysis including more than 3,500 participants from 59 studies across the globe indicated worse tear function scores among patients with DM than those without.^36^ These differences were maintained regardless of the type of diabetes and ethnicity.^36,37^ Thus, unlike in patients without DM, these data suggest that in patients with DM, decreased tear production and poor corneal sensitivity due to persistent hyperglycaemia-i nduced injury to the corneal receptors result in a more severe dry eye vicious cycle. This is corroborated by the results of this meta-analysis, which found no significant difference in tear function between patients with DM having good glycaemic control and participants without DM.^36^

## Screening of dry eye disease in patients with diabetes

Nearly 60% of patients with diabetes have one or more of the ocular complications, which progressively increase after 5 years of a diabetes diagnosis.^38^ However, as DED is a multifactorial disease, the presence of multiple risk factors (environmental factors such as digital screens, intrinsic factors such as hypertension and concomitant therapies increasing the DED risk) in addition to diabetes may prepone the occurrence.

A dry eye examination, as described in detail in the TFOS DEWS II Diagnostic Methodology report, should be added to the routine monitoring of patients with DM, at least in those at high risk.^2,39^

- All patients with diabetes should undergo comprehensive ocular screening for retinopathy, glaucoma and dry eye at least annually.
- More frequent screening is suggested in:
  - patients with long-standing diabetes and poor glycaemic control;
  - patients with a previous diagnosis of DR or neuropathy;
  - post-menopausal females with diabetes and those on oestrogen replacement therapy; and
  - patients with comorbidities such as hypertension, Parkinson’s disease and depression.

*Chronic hyperglycaemia leads to histological abnormalities in the lacrimal glands and corneal epithelium and interferes with the regulation of expression of certain mediators causing dysfunction of the lacrimal functional unit, resulting in dry eye disease.*

*AGEs = advanced glycation end-products; ALS2CL = ALS2 C-Terminal Like; APC = antigen-presenting cells; Apo = apolipoprotein; ARHGEF19 = Rho Guanine Nucleotide Exchange Factor 19; CAMs = cell adhesion molecules; CD = cluster of differentiation; IFN = interferon; IL = interleukin; KIAA1109 = FSA (fragile site-associated) protein; LFU = lacrimal functional units; MMP = matrix metalloproteinase; NF = neuronal fibres; PLXNA1 = Plexin A1; POLG = DNA polymerase subunit gamma; SIRT = sirtuin; TH = T-helper cells; WIPI1 = WD repeat domain, phosphoinositide interacting 1; ZMIZ2 = Zinc Finger MIZ-Type Containing 2.*

Diagnosis of DED is based on the use of a combination of subjective and objective assessment.^2^ The DEWS report recommends implementing one of the questionnaires for subjective evaluation in the clinical setting for screening purposes.^2^ Objective diagnostic methods have evolved over the years. Based on the availability and patient inclination, tests yielding specific information to aid early treatment should be preferred. Additional tests should be chosen such that they identify DED subtypes and guide the treatment strategy significantly. Once a DED diagnosis is confirmed, aqueous deficient dry eye and evaporative dry eye can be distinguished based on the results of relevant tests.

## Bidirectional association between dry eye disease and diabetes

The degree of tear function is governed by glycaemic control; therefore, glycaemic control in patients with DM is critical for maintaining tear function.^36^

## Clinical evidence: Impact of poor glycaemic control on dry eye disease

In patients with uncontrolled plasma glucose, dry eye symptoms were reported to be severe.^14^ A 12-month observational study in patients with diabetes found that the severity of DED (assessed by Ocular Surface Disease Index [OSDI] score) was linearly associated with A1C.^40,41^ The OSDI score was 4 for A1c 6.0–7.0%, 10 for A1c 7.1–8.0%, 22 for A1C 8.1–9.0% and 28 for A1C >9.0%.^40^ Overall, the OSDI score was less than 12 for patients with A1C less than 8.0% and greater than 12.0% for A1C greater than 8.0%. An open-l abel 6-week study found that in patients with severely uncontrolled plasma glucose (A1c >12.0%), the mean levels of OSDI score (>28.0), tear film osmolarity (TFO) measurement (>349.0 mOsm/L), tear breakup time (TBUT) test (>6.0 s) and Schirmer’s test (>8.0 mm) were considerably beyond the cut-offs.^42^ These values significantly reduced by approximately 10.0–40.0% when plasma glucose was reduced by 50.0% (fasting blood glucose [FBG] 300.0–153.0 mg/dL) and post-prandial blood glucose (PPBG; 431.0–252.0 mg/dL)] and HbA1c reduced by 25.0% (from 12.0 to 9.0%).^42^

In addition to the direct impact of poor glycaemic control on DED, microvasculopathy, such as retinopathy and neuropathy, which also occur as a consequence of long-standing hyperglycaemia, affects the severity of DED. Patients with reduced corneal sensitivity may remain asymptomatic and thus may not seek medical advice, leading to worsening of DED and increasing the risk of complications. Thus, patients with DED and either retinopathy or neuropathy due to prolonged hyperglycaemia also experience poor scores on DED parameters than those with DED alone.

Thus, poor glycaemic control, directly and indirectly, affects the severity of DED in patients with diabetes. Therefore, treatment of DED in patients with diabetes should focus not only on symptomatic relief, but also on glycaemic control.

## Impact of dry eye disease on glycaemic control

DED can cause loss of visual acuity, which may complicate diabetes self-management.^43^ Clinical studies and population-based surveys have shown that symptomatic DED impacts the quality of vision because of the abnormalities of the tear film and associated optical refracting surfaces and, consequently, decreases the ability to perform self-care activities.^44^ Among the various affected activities are cooking healthy food, exercising, monitoring blood glucose and taking insulin and medications that help patients maintain a stable plasma glucose level.^44^ It also affects the ability to navigate spaces, which could limit movement. Thus, DED in patients with diabetes could affect the entire self-care regimen. Consequently, reduced adherence to the use of medications prescribed for diabetes, as well as reduced physical activity, could negatively affect glycaemic control, which could, in turn, aggravate DED symptoms. Thus, patients with both DED and diabetes are at risk of a vicious cycle of negative health outcomes.^43^

## Prevention and treatment of dry eye disease

### Prevention of dry eye disease

Marked symptoms in the absence of clinically observable signs may indicate the possibility of neuropathic pain, which is prevalent in patients with diabetes. Therefore, DED signs alone may still warrant management to prevent DED manifestation.^45^ The prevention of DED involves the identification of risk factors, such as poor glycaemic control in patients with diabetes, educating patients regarding these and other environmental risk factors and managing based on DED severity.^5,8,30,46^

### Treatment of dry eye disease

The goal of DED management in diabetes is to re-establish homeostasis of the ocular surface by interrupting the vicious cycle of the disease. It also includes the use of sustainable alternatives to avoid a return to the pathophysiological cycle and re-emergence of symptoms. To achieve this successfully, it is essential to target all contributing factors and not rely only on tear replacement.^45,46^

### Lid hygiene management

Case–control studies conducted in India have shown a possibility that environmental factors, such as air pollution, wind, humidity and altitude, may affect the signs and symptoms of DED.^17^ Therefore, lid hygiene plays an important role in the management of DED. The lid hygiene management has been studied in various clinical studies described by DEWS. It includes eyelid warming, massaging and cleaning using a lid scrub, wipes, neutral shampoo or a combined lid cleanser and artificial tears. These studies have demonstrated improvement in signs and symptoms, TBUT and eyelid margin status.^41,46,47^

### Advantages

It helps to decrease dry eye symptoms, re-establish tear film stability, improve Meibomian gland (MG) secretion, increase tear film lipid layer thickness and reverse MG dropout.

### Limitations

Long-term treatment adherence is challenging due to patient-centred factors (compliance is 54%).^43,48^

Patient education plays an important role in the management of patients with both diabetes and DED (*Table 2*).

Both DED and DM are chronic, incurable disease conditions; hence, treatment should be individualized to ensure symptomatic relief, disease control and prolonged adherence. Available evidence corroborates that patients with DED, who require frequent dosing with lubricants, should avoid the use of preservative-containing benzalkonium chloride (BAK) ocular lubricants.^2^ Preservative-free drops may be a better choice, as these have demonstrated greater efficacy in patients with DED. Drops preserved with BAK are no longer recommended (*Table 3*).^2^

The various therapeutic agents available for the treatment of DED are listed in *Table 4*.^49–67^

## Algorithm for the management of dry eye disease in patients with diabetes

**Figure 2:**
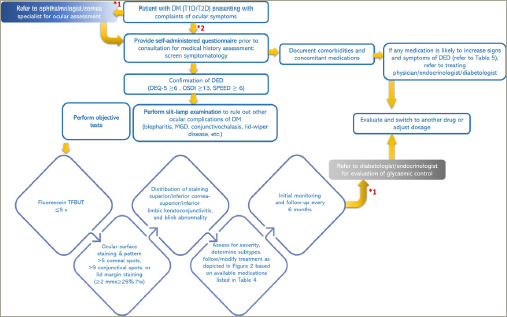
Algorithm for the management of patients with diabetes and dry eye disease

**Figure 3:**
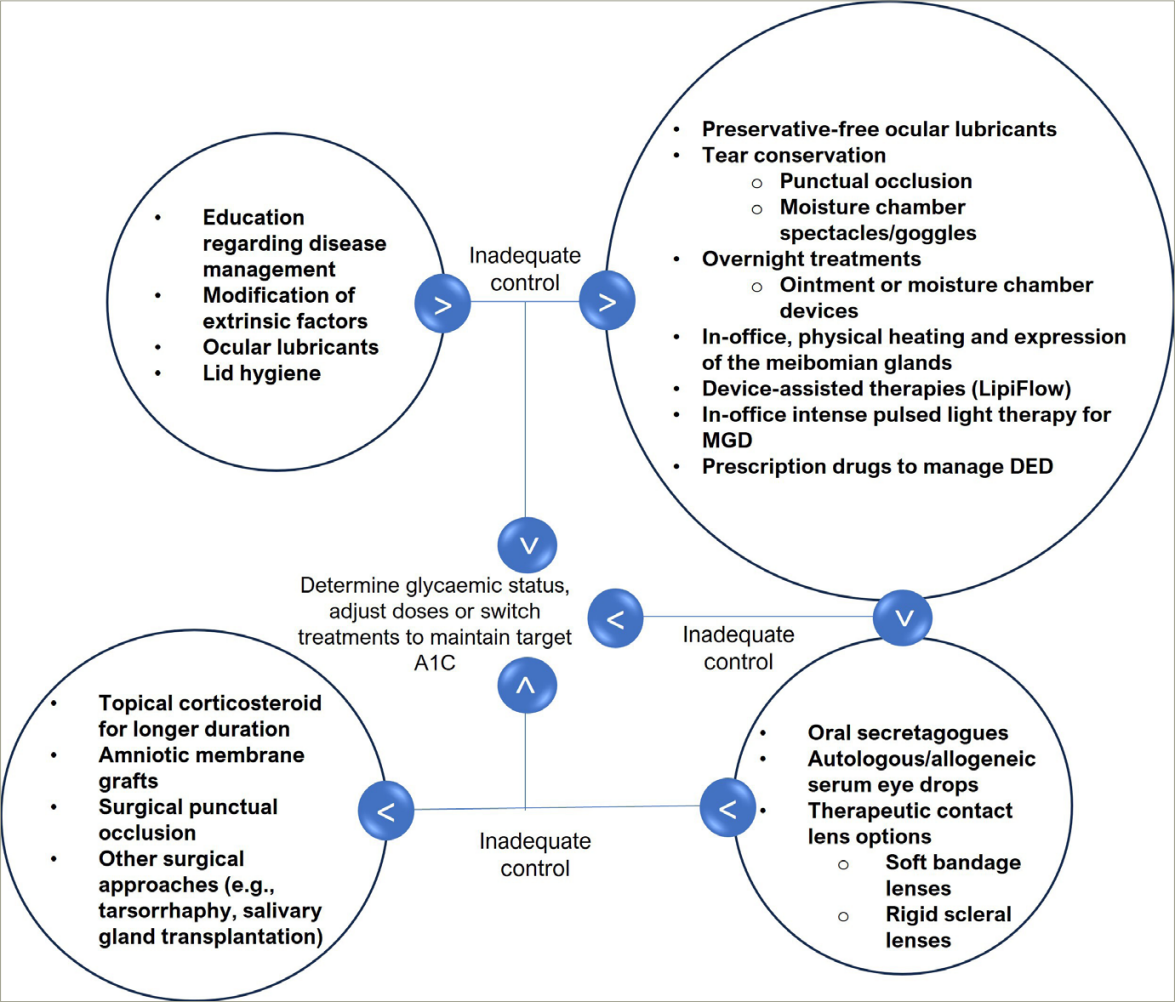
Dry eye disease in patients with diabetes: Modification sequence of treatment modalities based on response to treatment

Based on the methods of DED management described in respective sections and various guidelines, an algorithm (*Figure 2*) specifically for patients with diabetes and DED may be useful.^1,2,5^ The treatment modalities should be adjusted/modified based on the response to treatment initiated as per disease severity (*Figure 3*).

## Pharmacotherapy of diabetes: Effect on dry eye disease

The fundamental approach of T2D management has been progressing from glycaemic control to organ protection. The ocular therapeutic or adverse effects of antidiabetic drugs should be of concern when these are to be chosen for patients with chronic conditions such as DED.^68^

**Table 2:** Patient education: Dry eye disease in diabetes

Dry eye disease	Diabetes and other risk factors
The patient should be educated and reassured that even though DED is chronic and not curable, it can be well controlled with appropriate treatment and does not generally lead to loss of vision	Patients should be educated on the association between glycaemic control and severity of DED and other ocular complications and therefore encouraged to remain adherent to lifestyle changes and pharmacotherapy for both DED and diabetes
Appropriate instruction regarding the use of medications should be provided	
The patients should be informed about any possible irritation or stinging with ophthalmic medications, the time required to relieve symptoms and the importance of adherence for maintaining control of symptoms	
Therapeutic alternatives should be tailored to the patient’s needs and ocular comfort	Patients should avoid or minimize exposure to aggravating factors, such as air conditioners, low-humidity environments, contact lenses and digital screens
Patients should be cautioned about the increase or persistence of DED symptoms for almost a year after post-ocular procedures, such as LASIK or refractive surgeries	Patients should be advised for periodic (6 months) follow-up ophthalmic examinations even if DED symptoms are controlled, as they may become asymptomatic with increased age or loss of corneal sensitivity

*DED = dry eye syndrome; LASIK = laser-assisted* in situ *keratomileusis.*

**Table 3:** Types of dose containers based on units and use of preservatives^2^

Type	Feature	Limitation	Implication(s)
Multidose artificial lubricants with preservatives	Economic versus unit dose	Contains preservatives	Greater risk of adverse changes to the ocular surface and toxicity
Dispensers with unidirectional valves	Multidose bottles yet preservative-free	Expensive Availability issues	Lesser risk of adverse events May be less commonly used
Unit dose, preservative-free	Absence of preservatives	Expensive More difficult for less dextrous individuals to open	Lesser risk of adverse changes to the ocular surface and toxicity
Disappearing preservatives containing multiple dose drops: **Oxidative preservatives** Sodium chlorite Sodium perborate Polyquaternium-1	Sodium chlorite decomposes into chloride ions and water when exposed to UV light after instillation, and sodium perborate decomposes into water and oxygen in contact with the tear film	May have negative effects on the ocular surface	Avoid issues with long-term exposure to preservatives Lower impact on the ocular surface

*UV = ultraviolet.*

Most classes of antidiabetic agents do not demonstrate anticholinergic effects or a direct association with dry eye. However, some increase the risk of macular oedema and/or worsen DR. As the presence of both macular oedema and retinopathy double the risk of experiencing DED, patients with both DED and diabetes should be monitored for ocular signs and symptoms, or an alternative oral hypoglycaemic agent should be prescribed.^26,27^ Some antidiabetic drug classes have also shown improvement in DR.^69–72^ However, except metformin, none of these drugs has been reported to directly increase the incidence of dry eye signs or symptoms.^69–72^

### Association of older antidiabetic agents with dry eye disease

#### Biguanide, thiazolidinediones and sulfonylureas

In patients with T2D, usually, the first-l ine pharmacotherapy is metformin. A case–control study found that metformin use was the only independent factor associated with dry eyes.^69^ The probable underlying mechanism could be the anticholinergic effect of metformin, particularly in high doses and frail patients.^70^ However, metformin has demonstrated a protective effect against DR.^71,72^

Fluid retention occurs in 5–15% of patients prescribed thiazolidinediones (TZD: pioglitazone).^73^ It may result in the development of macular oedema, which is a complication of DR.^74^ The cessation of TZDs results in the resolution of macular oedema, as well as peripheral oedema.^73^ These factors should be considered, and alternate options should be chosen when assessing treatment options for patients with diabetic macular oedema (DME), especially those with concomitant peripheral oedema.^74^ Interestingly, switching from TZDs to gliclazide has shown improvement in the visual acuity of a patient with bilateral macular oedema.^75^ Indeed, studies have shown that gliclazide prevents the progression of DR, particularly the worsening of proliferative diabetic retinopathy (PDR), better than glibenclamide.^76,77^ Glibenclamide has been reported to affect lens changes due to osmotic effects, resulting in reduced visual acuity.^78^ Therefore, in diabetic patients diagnosed with DED, the benefit of using TZD or glibenclamide should be weighed against the risk, as DED also reduces visual acuity.^43^

### Association of newer antidiabetic agents with dry eye disease

#### Glucagon-like peptide 1 receptor agonists (incretins)

Initial screening studies and case reports found that glucagon-l ike peptide 1 receptor agonist (GLP-1 RA) therapy led to a brief worsening of DR, which subsequently improved with continued treatment.^79,80^ Few cases of complete reversion of DME with exenatide have been reported.^81^ However, cases of DR worsening to bilateral PDR with improved glycaemic control with exenatide have also been documented.^82^ Patients treated with semaglutide were found to experience higher incidences of retinopathy complications (vitreous haemorrhage, blindness or conditions requiring intravitreal treatment or photocoagulation).^83^ It has been argued that patients with pre-existing maculopathy, severe grade of retinopathy and long-standing diabetes may increase susceptibility to sustained deterioration of retinopathy.^80^

#### Dipeptidyl peptidase-4 inhibitors (gliptins)

Case reports have demonstrated a reduction in the rate of DR with dipeptidyl peptidase-4 (DPP-4) inhibitors; nevertheless, their use for less than a year may lead to an early progression of DR.^84,85^ Lately, DPP-4 inhibitors (saxagliptin and vildagliptin) have been associated with the manifestation of cicatricial pemphigoid, a toxic reaction to drugs that leads to severe dry eye.^86^ Abnormal scarring is the hallmark of mucous membrane pemphigoids lesions heal via a fibrosing process, resulting in cicatricial lesions that can cause severe impairment of the eyes. Gliptins are probably responsible for some mucous membrane pemphigoids. In addition, as cicatricial pemphigoid was first reported at 4 weeks and 36 weeks of gliptin use, periodic ocular monitoring/assessment should be performed. Therefore, when prescribing gliptins to patients with diabetes with concomitant DED, the risk versus benefit should be carefully evaluated. These cases also underscore the importance of taking into account the medical history, including concomitant medications prescribed by other treating physicians.

#### Sodium-glucose co-transporter 2 inhibitors

Evidence from studies suggests that sodium-glucose co-transporter 2 inhibitors (SGLT2i) may confer retino-protective effects by targeting pathways promoting inflammation, oedema and retinal pathological changes, resulting in vision abnormalities. Thus, SGLT2i use could limit disease progression and reduce drug burden in patients with high-risk T2D and DR.^87^ A large retrospective cohort analysis found that compared with GLP-1 RA, the use of an SGLT2i was associated with a 22% lower risk of DED, although the reduction in A1C was not significantly different.^37^ Therefore, SGLT2 inhibitors are protective against DED independent of its anti-hyperglycaemic effects and may also reduce its progression.

Newer antidiabetic agents may thus provide an important locus for clinical choices about prescribing diverse antidiabetic medications to interrupt or avert DED in patients with T2D.

Nevertheless, reno-retinal dissociation in therapeutic outcomes has been noted with empagliflozin similar to GLP-1 agonist liraglutide, Angiotensin converting enzyme (ACE) inhibitor ramipril and perindopril and diuretic indapamide. In studies with these agents, renal outcomes showed remarkable improvement, although improvement in retinal outcomes was lacking.^88^

**Table 4:** Pharmacological management of dry eye disease in patients with diabetes mellitus^49–67^

Drug class (route)	Mechanism, purpose	Limitations include adverse reactions	Advantages	Effect on clinical outcomes	Place in therapy	Interaction with antidiabetic therapy/Effect on glycemic control
1. Tear substitutes (topical) HA, HPMC, CMC, PEG, PG^49^	↑ tear fluid clearance ↓ pro-inflammatory factors ↑ Osmoprotection autophagy	Only symptomatic benefit, no effect on the pathology	Easy to use Available in a wide range of formulations Low risk-profile	Combination formulations are more effective than single active ingredient artificial tears. PEG-containing more effective versus CMC-and HPMC-containing	First-line Mild DED	None reported
a. Aqueous-based (contains osmotic agents, osmoprotectants, antioxidants, preservatives and inactive)	Target the muco-aqueous phase of the tear film			Improve symptoms related to all subtypes of DED	First-line	None reported
b. Lipid-based (mineral oils and phospholipids; drops, nano-emulsion drops, or liposomal sprays)	Target the superficial tear lipid layer (MGD)			Predominantly more effective in evaporative dry eye Water-free formulation-preservative-free available	Use when aqueous-based drops are not effective	None reported
c. Mucin-based (trehalose)^50^	Maintains cell protein integrity during drying and rehydration, and it has been shown to protect against oxidative strain and stabilize protein function		Rehydrate tear film by retaining moisture when drying out and aid in increasing its thickness Protect against future irritation by improving corneal staining and protecting corneal epithelial cells from apoptosis after desiccation Support homeostasis of tear film by restoring osmotic balance to ocular surface and maintaining homeostasis of corneal cells	In ophthalmic products, trehalose enhances active ingredients (e.g. carboxymethyl cellulose) to help: Protect corneal cells from desiccation p Restore osmotic balance to the ocular surface Maintain the homeostasis of corneal cells Increased tear film thickness after instillation of one trehalose-containing drop up to 240 min compared with drops without trehalose. Better patient satisfaction and a therapeutic advancement in treatment of moderate to severe DED when comparing an eyedrop containing hyaluronic acid-trehalose with an HA-only eyedrop	Proposed as first-line	None reported
d. Biological (autologous or allogeneic serum [AS], cord blood serum [CBS], autologous platelet lysate [APL], and platelet-rich plasma [PRP])^51^	Improve tear stability, fluorescein, and rose bengal staining scores, as well as subjective symptom scores	Low/very low certainty of the evidence More likely to produce ocular adverse events versus with artificial tears, but not statistically significant Risk of contamination, denaturation with time Needs blood sample collection and time for preparation time	Biological tear substitutes were the most effective inten/entions combinations of topical secretagogues and artificial >monotherapy of topical secretagogues. Beneficial effect on advanced and severe cases	APL: a most effective treatment for lowering ocular surface disease index PRP: most reduction in corneal fluorescein staining scores AS significantly improved OSDI scores vs artificial tears^51^ APL and PRP significantly decreased corneal fluorescein staining more than AS^51^ AS significantly improved TBUT compared with artificial tears^51^ Only AS significantly increased TBUT compared with PRP. CBS: most effective for increasing TBUT Eledoisin: superior to others in improving Schirmer scores	Severe DED	None reported
2. Immunomodulatory						
a. Corticosteroids (topical) e.g. dexamethasone, fluoromethoIone, loteprednol methylprednisolone, prednisolone, hydrocortisone systemic^52,53^	Anti-inflammatory actions on multiple targets: including decreasing expression of cytokines, maintaining the integrity of corneal epithelium	Not for chronic use. Possible side effects Increased risk of cataracts and IOP	Fast action, highly effective	Small to moderate effect on lowering corneal staining scores and relief of symptoms^52^ May TBUT slightly^52^ b No effect on tear osmolarity^52^ 5-fold f risk of IOP elevation versus lubricants^52^ Small to moderate degrees of symptom relief versus CsA^52^ Long-term use can present complications such as ocular hypertension and opportunistic infections^54^'^55^	Conservative, second-line Moderate DED Long-term use in Severe DED Systemic corticosteroids only in very severe diseases for a short duration with continuous glycemic monitoring	Case reports of increased requirements of high insulin doses with topical corticosteroids for skin/oral mucosal conditions, especially in the elderly. 24% greater odds of risk of developing DM, Risk is not potency dependent but related to cumulative factors) dose and duration of use)^55^
b. Cyclosporine A oil-based and aqueous emulsions^55^	Anti-inflammatory and immunosuppressive properties by preventing T-cell activation and inflammatory cytokines production	Ocular burning Delayed achievement of full therapeutic effect Low bioavailability Reports of high patient discomfort and dissatisfaction	Long-term use	Improves tear film stability ↓ recurrent corneal erosions Completely heals areas of previous epithelial loss Reduced inflammation leads to improves mechanical stress and epithelial integrity, resulting in a reduction of signs and symptoms Improves corneal staining, Schirmer's score, TBUT, and subjective patient symptoms improved in 50% of patients Use in combination with a topical corticosteroid may reduce irritation Demonstrated early improvement of TBUT and patient compliance, than diquafosol^56^	Second-line: Use in those refractory to ocular lubricants and lid hygiene Moderate DED	None reported
c. Lifitegrast^57^	Novel-integrin antagonist Inhibits lymphocyte activation by blocking ICAM-1 and LFA-1 receptors	Site irritation, dysgeusia, ↓ visual acuity, blurred vision, conjunctival hyperemia, eye irritation, headache, increased lacrimation, eye discharge, eye discomfort, eye pruritus, and sinusitis	No significant systemic absorption, so there is negligible risk of drug interactions	OPUS-1-4 Statistically significant improvements in inferior corneal fluorescein staining scores and eye dryness scores versus placebo	Second-line Use in patient's refractory to artificial tears Moderate DED	None reported Indirect: causes dysgeusia which may lead to reduced food intake and weight loss and may cause difficulty in maintaining glycemic control
3. Antibiotics						
a. Doxycycline (topical/oral)	↓MMP-9 expression, macrophage, ↓IL-1β, ↓IL-6, ↓TNF-α	Dermatologic and gastrointestinal complications, hypersensitivity More side-effects anorexia, nausea, vomiting, diarrhoea, rash, photosensitivity, urticaria, and hemolytic anaemia	Break the vicious cycle of impaired meibum consistency, bacterial infection and toxin production, inflammation, and tear film instability	All signs of eyelid margin disease improved Significant improvement in TBUT Less effective than azithromycin^58^	Second-line Moderate DED MGD	Experimental study: improves glycemic control at low dose^59^
b. Azithromycin (oral)	Restores the levels of carotenoids in meibum	Nausea, diarrhoea, abdominal cramp, decreased appetite, rash, pruritus, photosensitivity, and angioedema		Improves VA, conjunctival redness, and corneal staining. More effective than doxycycline: More patients switched to azithromycin from doxycycline (17% versus 67%; p<0.005) Values of signs and symptoms were significantly better for azithromycin than for doxycycline. Fewer side-effects To be used with caution in patients with CVD	Second-line moderate DED MGD	Modest improvement in glycemic control^60^
4. Other						
a. Omega three fatty acids^61,62^	↓activation of pro-inflammatory cytokines ↑ anti-inflammatory PGs Promotes the resolution of inflammation via resolvins and improves neuroprotection via neuroprotectants	Inadequate evidence for clinical efficacy. Meta-analysis demonstrates heterogeneity of studies. Effective primarily at high doses Not cost-effective^63^	Limited benefits are reported as described in the adjacent column	Smaller RCTs suggest statistically significant benefits in symptom scores (TBUT significantly greater by 1.58 s and improves Schirmer's test but no effect on OSDI) that are not always clinically relevant.^61,62^ Several studies support oral supplementation: the daily dose, duration of its intake and percentage of eicosapentaenoic acid (EPA) have demonstrated a significant positive correlation with a reduction in DED symptom scores.^63,64^ Currently, artificial tear drops containing omega-3 fatty acids are available.^65,66^ The eye drop containing omega-3 fatty acids may increase lipid layer thickness and also improve DED signs and symptoms in evaporative DED^65,66^	Moderate DED	Improves glycemic control^62^
b. Mucin secretagogues (topical) e.g. diquafosol and rebamipide	To reduce tear deficiency: P2Y2 purinergic receptor agonist stimulates conjunctival epithelial cells for water secretion and conjunctival goblet cells for mucin secretion. Stabilize the tear film and repair the corneal epithelial damage Increase tear secretion	Eye discharge, itching, or irritation^67^	Benefits in terms of stabilising the tear film and repairing the corneal epithelial damage^67^	Combination of artificial tears and diquafosol sodium: Increase the wettability time of the ocular surface and the therapeutic effect of diquafosol Corneal fluorescein staining scores: Diquafosol better than artificial tears TBUT: diquafosol plus artificial tears, rebamipide plus artificial tears, rebamipide, diquafosol better than eledoisin Schirmer scores: eledoisin >diquafosol > artificial tears	Second-line moderate DED	None reported with either drug

*APL = autologous platelet lysate; AS = autologous or allogeneic serum; CBS = cord blood serum; CMC = carboxymethyl cellulose; CsA = cyclosporine A; DED = dry eye disease; EFA = eicosapentaenoic acid; HA = hyaluronic add; HPMC = hydroxypropyl methylcellulose; ICAM = intercellular adhesion molecule; IL = interleukin; IOP = intraocular pressure; LEA-1 = lymphocyte function-associated antigen-1; MGD = Meibomian gland dysfunction; MMP = matrix metalloproteinase; OPUS-1-4 = Ocular Protection and Utilization Study; OSD! = ocular surface disease index; PEG = polyethylene glycol; PG = propylene glycol; PRP = platelet-rich plasma; P2Y2 = P2Ypurinoceptor 2; RCT = randomized controlled trial; TBUT = Tear breakup time; TNF = tumour necrosis factor; YA = visual acuity.*

*1 = if the patient initially presented to primary care physicians; *2 = if the patient presents to an ophthalmologist/cornea specialist.

DED = dry eye disease; DEQ-5 = dry eye questionnaire; DM = diabetes mellitus; l*w = length X width; MGD = meibomian gland dysfunction; mm = millimeters; OSDI = Ocular Surface Disease Index; s = seconds; SPEED = Standard Patient Evaluation of Eye Dryness Questionnaire; T1D = type 1 diabetes; T2D = type 2 diabetes; TFBUT = tear film break up time; x = multiplication.

#### Insulin therapy

Sporadic cases have reported the association of intensification of insulin therapy with diabetic papillopathy.^89^ It has been linked with rapid glycaemic control in diabetic patients without a history of retinopathy.^89^ Topical insulin helps acinar cells proliferate and accelerate the restoration of the lacrimal gland by stimulating the insulin-l ike growth factor-1 receptor. A study is underway to determine the effects of topical insulin on tear inflammatory mediators interleukin-1a, interleukin-6 and matrix metalloproteinase-9 in people with diabetes and DED.^90^ A trial with topical insulin has demonstrated significant and similar improvement in the OSDI score as artificial tears; however, long-term studies in a larger patient population are required to confirm the efficacy and safety of topical insulin.^91^

*A1c = glycated haemoglobin; DED = dry eye disease; MGD = meibomian gland dysfunction.*

**Table 5:** Agents to treat comorbidities in diabetes: Proposed to increase risk or worsen dry eye syndrome^92–96^

Adrenergic blockers (atenolol, metoprolol and labetalol)
Thiazide diuretics (indapamide, hydrochlorothiazide and metolazone)
Antiparkinsonian agents (levodopa, benztropine and pemipexole)
COPD (glycopyrronium, aclidinium and umeclidinium)
Antidepressants (amitriptyline, bupropion, duloxetine and fluoxetine)

*COPD = chronic obstructive pulmonary disease.*

Therefore, antidiabetic agents with negligible ocular adverse effects in patients with or at risk of DED, macular oedema or DR should be preferred. If there is a need to use agents known to negatively affect signs and symptoms or pathology of DED, periodic monitoring should be performed, and the patient should be made aware of possible worsening of symptoms. As metformin is the predominant first choice in most patients with a new diagnosis of diabetes, and is used for a prolonged duration, periodic monitoring is necessary for those at risk of DED (severe retinopathy and macular oedema).

## Pharmacotherapy for common comorbidities in patients with diabetes and association with dry eye disease

Hypertension (73%), Parkinson’s disease (38–71% greater risk), chronic obstructive pulmonary disease (COPD; 18 versus 10%) and depression (two-to threefold higher) are some of the common comorbidities in patients with diabetes.^92–96^ Some of the agents used to treat the comorbidities are listed in *Table 5*.^92–96^

Chronic use of these drugs can increase the risk or aggravate pre-existing DED. The age-adjusted 10-year incidence of dry eye in people using these drugs is greater compared with the general population.^97^ Therefore, to avoid any unwarranted exacerbation of DED, it is necessary to obtain an adequate medical history of comorbidities and concomitant medications before prescribing any new agent, even if the patient is treated by different specialists.

## Conclusion

DED and diabetes may frequently coexist. Although DED is a non-l ife-threatening condition, it tremendously affects the quality of life of the individual. Besides, it may negatively impact the management of diabetes, while the latter may increase the severity of DED and the risk of complications. Hence, appropriate management of DED and diabetes in such patients is necessary. Physicians who treat patients with diabetes should be able to administer DED questionnaires as a screening tool. Antidiabetic agents with negligible ocular adverse effects in patients with or at risk of DED, macular oedema or DR should be preferred. If there is a need to use agents known to negatively affect signs and symptoms or pathology of DED, periodic monitoring should be performed, and the patient should be educated regarding the probable impact.

It is imperative to address the risk of a vicious cycle of poor glycaemic control and worsening DED by considering all pertinent factors. Therefore, this review describes screening, diagnosis and treatment preferences that are an invaluable resource for physicians managing patients with both conditions. A coordinated approach to managing patients with DED and diabetes is necessary, as both conditions can significantly impact the patient’s prognosis.
